# Editorial: Intermittent Hypoxia: From Basic Mechanisms to Clinical Insights and Therapeutics

**DOI:** 10.3389/fneur.2020.00647

**Published:** 2020-08-21

**Authors:** Lena Lavie

**Affiliations:** The Ruth and Bruce Rappaport Faculty of Medicine, Technion Israel Institute of Technology, Haifa, Israel

**Keywords:** intermittent hypoxia, obstructive sleep apnea, co-morbidities, oxidative stress, inflammation, endothelial dysfunction

The implications of various forms of hypoxia to physiological or pathological conditions are enormous. In this manuscripts' collection of “Research Topics,” expert authors focused on the effects of intermittent hypoxia (IH), the hallmark of obstructive sleep apnea (OSA) on physiological and pathophysiological conditions and potential treatments, each, contributing original research articles and reviews in their field.

OSA is a major health risk-factor, depicting increased mortality rates. This is attributed to its high occurrence and association with various co-morbidities, including cardiovascular, cerebrovascular, metabolic, and increased cancer risk. Thus, research has been aimed at identifying the underlying mechanisms for these associations. Two of the most fundamental mechanisms, oxidative stress and inflammation were shown to be affected by IH. Yet, different severity-dependent changes in frequency, magnitude, and chronicity, considerably alter the patterns of IH, and thereby initiate diverse and sometimes opposing outcomes, altering physiological measures, multiple signaling pathways, and genetic-epigenetic expression.

Although the association with various co-morbidities is evident, it is also evident that many OSA patients are free of co-morbidities. Some paradigms of IH in human, animal, and *in-vitro* studies, showed beneficial rather than detrimental effects, suggesting that some IH patterns may induce protective mechanisms to tissues and organs and particularly the heart, rather than damage. The involvement of oxidative stress through reactive oxygen species (ROS)-dependent signaling pathways is implicated both in IH and in OSA. Thus, while some patterns of IH are detrimental, others could confer protection (1).

Mendelson et al. systematically reviewed physical activity and exercise interventions in OSA patients. Sedentary life, a characteristic of OSA, is attributed to fatigue, somnolence, and obesity, and consequently, to decreased physical activity. A meta-analysis of all studies reporting on objectively measured physical activity in OSA patients explored the effects of physical training on apnea severity, Epworth sleepiness scale, BMI and cardiorespiratory fitness measured by VO_2_ peak. They confirmed that OSA was associated with significantly lower levels of objectively measured physical activity, and demonstrated that structured exercise training decreased OSA severity, improved daytime sleepiness and cardiorespiratory fitness, without affecting BMI.

Evidence linking OSA with essential hypertension has been reported ever since OSA has been scientifically investigated. Joyeux-Faure et al. investigated the association between resistant hypertension, that is, patients whose blood pressure remains elevated in spite of concurrent use of three antihypertensive medications of different classes, and OSA. They showed that this association is characterized by a higher burden of metabolic syndrome, higher creatinine levels, and that adherence to continuous positive airway pressure (CPAP) treatment reduced night-time but not daytime blood pressure.

Several papers highlighted mechanisms involved with cardiovascular complications in OSA. Gabryelska et al. reviewed the involvement of platelets in the development of cardiovascular morbidity in OSA. Blood platelets play a central role in homeostasis and thrombosis. As such, their activation and function considerably contribute to cardiovascular morbidity. Although the studies focusing on platelet function in OSA and IH are limited, this review is comprehensive and systematic. Increased mean platelet volume (MPV), indicative of cardiovascular morbidity, was associated with OSA severity. The efficacy of CPAP treatment in decreasing platelet-dependent cardiovascular risk, based on platelet activation markers, is summarized, while anti-platelet treatments combined with CPAP are suggested as potential therapeutic targets to decrease cardiovascular risk in OSA.

The significance of IH-dependent ROS production to vascular health was investigated by Avezov et al.. Endothelial Cell-Colony Forming Units (EC-CFUs) and their angiogenic paracrine properties were shown to closely correlate with vascular functions and retain measures of vascular health (2). Patients after acute myocardial infarction (AMI) and concomitant sleep disordered breathing (SDB) had higher EC-CFUs numbers and higher paracrine angiogenic capacities than AMI patients without OSA (3). Similarly, in healthy subjects, IH *in-vitro* contributed to increased EC-CFUs numbers, their angiogenic capacities, ROS production and vascular endothelial growth factor (VEGF). Treatment with antioxidants and ROS inhibitors decreased or abolished EC-CFUs numbers and angiogenic measures, emphasizing the crucial role of ROS in mediating vascular health, possibly by activating vascular adaptive-protective mechanisms.

The association between IH and cardiovascular disorders consequent to increased endothelial dysfunction was investigated in healthy subsects exposed to IH, using exosomal release in plasma and its potential control by drugs. Exosomes may act as vehicles for propagating damage to endothelial cells, while drug interventions may provide protection for the endothelium. Khalyfa et al. investigated the beneficial effects of celecoxib (CEL, COX-2 inhibitor) and losartan (LOS, angiotensin II receptor-1 antagonist) on IH-induced vascular dysfunction. The cardiovascular protective impact of LOS and CEL was mediated by their direct effects on endothelial cells, rather than modulating exosomal cargo.

A mechanistic approach was undertaken by Almendros et al. to investigate hypoxia inducible factor (HIF)-1α and VEGF expression in cutaneous melanoma (CM) patients with OSA. Recent epidemiological studies show an association between OSA and poorer solid malignant tumor outcomes. Relying on a large multicenter cohort of patients diagnosed with CM and SDB, they investigated putative pathways to explain these associations. HIF-1α and VEGF were determined in CM tumoral lesions. HIF-1α was independently associated with SDB severity. These findings shed a new light on the epidemiological association between cancer and OSA, whereby OSA may play a deleterious role in cancer outcomes. It has yet to be defined which types of cancers are more prone to express poorer outcomes in OSA.

Alzheimer's disease (AD) is another pathology associated with OSA. Evidence from patients and animal models suggests that OSA may increase the risk of AD and that AD is associated with reduced brain tissue stiffness. Menal et al. investigated whether IH altered brain cortex tissue stiffness in early-onset AD mutant mice exposed to IH mimicking OSA. Chronic IH did not modify the stiffness of brain cortex in normal mice, and similarly did not alter the mechanical properties of the cortex in this murine AD model. AD mutant mice exhibited reduced brain tissue stiffness following both normoxia and IH, likely due to increased edema and demyelination in AD.

Collectively, the pathologies described herein are associated with OSA and IH. However, importantly, the IH in OSA is implicated in eliciting both detrimental and protective mechanisms in cardiovascular morbidity.

## Author Contributions

The author confirms being the sole contributor of this work and has approved it for publication.

## Conflict of Interest

The author declares that the research was conducted in the absence of any commercial or financial relationships that could be construed as a potential conflict of interest.
